# An electronic health record (EHR) phenotype algorithm to identify patients with attention deficit hyperactivity disorders (ADHD) and psychiatric comorbidities

**DOI:** 10.1186/s11689-022-09447-9

**Published:** 2022-06-11

**Authors:** Isabella Slaby, Heather S. Hain, Debra Abrams, Frank D. Mentch, Joseph T. Glessner, Patrick M. A. Sleiman, Hakon Hakonarson

**Affiliations:** 1 The Center for Applied Genomics, Children’s Hospital of Philadelphia, Philadelphia, PA 19104 USA; 2 Department of Pediatrics, Perelman School of Medicine, University of Pennsylvania, Philadelphia, PA 19104 USA

## Abstract

**Background:**

In over half of pediatric cases, ADHD presents with comorbidities, and often, it is unclear whether the symptoms causing impairment are due to the comorbidity or the underlying ADHD. Comorbid conditions increase the likelihood for a more severe and persistent course and complicate treatment decisions. Therefore, it is highly important to establish an algorithm that identifies ADHD and comorbidities in order to improve research on ADHD using biorepository and other electronic record data.

**Methods:**

It is feasible to accurately distinguish between ADHD in isolation from ADHD with comorbidities using an electronic algorithm designed to include other psychiatric disorders. We sought to develop an EHR phenotype algorithm to discriminate cases with ADHD in isolation from cases with ADHD with comorbidities more effectively for efficient future searches in large biorepositories. We developed a multi-source algorithm allowing for a more complete view of the patient’s EHR, leveraging the biobank of the Center for Applied Genomics (CAG) at Children’s Hospital of Philadelphia (CHOP). We mined EHRs from 2009 to 2016 using International Statistical Classification of Diseases and Related Health Problems (ICD) codes, medication history and keywords specific to ADHD, and comorbid psychiatric disorders to facilitate genotype-phenotype correlation efforts. Chart abstractions and behavioral surveys added evidence in support of the psychiatric diagnoses. Most notably, the algorithm did not exclude other psychiatric disorders, as is the case in many previous algorithms. Controls lacked psychiatric and other neurological disorders. Participants enrolled in various CAG studies at CHOP and completed a broad informed consent, including consent for prospective analyses of EHRs. We created and validated an EHR-based algorithm to classify ADHD and comorbid psychiatric status in a pediatric healthcare network to be used in future genetic analyses and discovery-based studies.

**Results:**

In this retrospective case-control study that included data from 51,293 subjects, 5840 ADHD cases were discovered of which 46.1% had ADHD alone and 53.9% had ADHD with psychiatric comorbidities. Our primary study outcome was to examine whether the algorithm could identify and distinguish ADHD exclusive cases from ADHD comorbid cases. The results indicate ICD codes coupled with medication searches revealed the most cases. We discovered ADHD-related keywords did not increase yield. However, we found including ADHD-specific medications increased our number of cases by 21%. Positive predictive values (PPVs) were 95% for ADHD cases and 93% for controls.

**Conclusion:**

We established a new algorithm and demonstrated the feasibility of the electronic algorithm approach to accurately diagnose ADHD and comorbid conditions, verifying the efficiency of our large biorepository for further genetic discovery-based analyses.

**Trial registration:**

ClinicalTrials.gov, NCT02286817. First posted on 10 November 2014. ClinicalTrials.gov, NCT02777931. First posted on 19 May 2016. ClinicalTrials.gov, NCT03006367. First posted on 30 December 2016. ClinicalTrials.gov, NCT02895906. First posted on 12 September 2016.

**Supplementary Information:**

The online version contains supplementary material available at 10.1186/s11689-022-09447-9.

## Introduction

ADHD is the most prevalent neurodevelopmental disorder in children [1, 2]. It is currently defined by persistent patterns of inattention and/or hyperactivity/impulsivity inconsistent with one’s developmental level, with symptoms usually continuing across the life span and resulting in impairments in social, educational, and work activities [3]. It is formally diagnosed by meeting the Diagnostic and Statistical Manual of Mental Disorders (DSM) criteria; however, physicians may diagnose patients based on symptoms and/or a trial of stimulant medication [4].

Because ADHD exists on a spectrum and presents in various forms [3, 5], patients may undergo a diagnostic odyssey before an ADHD diagnosis is reached. Moreover, ADHD frequently presents with various comorbidities, and it may be unclear whether impairments are due to the comorbidity or ADHD [6–8]. Comorbid conditions increase the likelihood for a more severe and persistent course of ADHD [7, 8] and may complicate clinical presentation and appropriate treatment choice [9, 10]. Due to this, developing an accurate algorithm to search for ADHD phenotypes in hospital-based EHR has proven challenging. Few articles describe the algorithms for ADHD, and they are for patient cases with ADHD exclusively or lack clarity on other psychiatric diagnoses exclusion [11–13]. A paucity of EHR algorithms for comorbid disorders also complicates creating an efficient and accurate algorithm.

Robust phenotype algorithms typically use empirical and/or machine learning algorithms, combining multiple data sources to achieve high positive predictive values (PPVs) in identifying cases and controls [14]. A thorough search and analysis of structured EHR data can account for systemic complexities such as changing ICD codes or disorder nomenclature. Numerous algorithms have used natural language processing (NLP)-based techniques as simple as using text words or as complex as utilizing comprehensive NLP tools and datasets for mining unstructured clinical data [15, 16].

Given the prevalence of comorbidities with ADHD, we believe an algorithm including comorbidities would be more representative of the patient population and permit a greater yield of ADHD cases within a large database. To address this, we developed a multi-source/multi-approach EHR rule-based algorithm with NLP text mining allowing for a more complete extraction of the patient’s medical record. Chart abstractions were completed to add evidence and assess confidence in the ADHD and comorbid diagnoses. Our objective was to create and validate an EHR-based algorithm to classify ADHD and comorbid psychiatric status in a pediatric healthcare network to be used in future genetic analyses and discovery-based studies. The underlying genetic etiology may be different for children with comorbid psychiatric conditions versus ADHD in isolation. We believe these cases represent a group of subjects that may be less responsive or amenable to traditional ADHD medications and may represent a suitable cohort for testing new non-stimulant compounds for ADHD.

## Methods

### Algorithm development

**Fig. 1:**
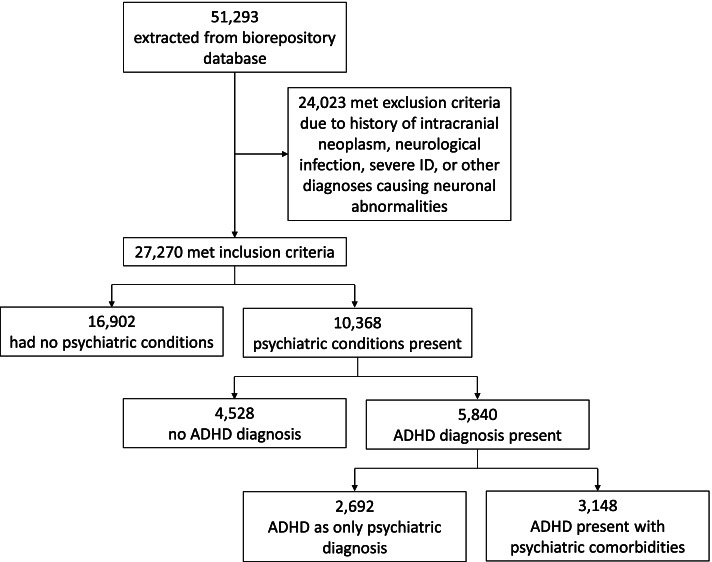
Concept overview of ADHD with comorbidities algorithm. Details for cases and controls are outlined in methods and supplementary tables

The rule-based phenotyping algorithm was developed using data from the CAG pediatric biorepository database at CHOP (Fig. 1). We extracted subjects genotyped on a genome-wide chip and with phenotype data available from 2006 to 2019. Recruitment was not targeted; the biorepository broadly reflects the incidence rates for pediatric disease in the USA, albeit enriched for several rare and specialty cohorts [17–19].

The algorithm for detecting psychiatric phenotypes draws information from multiple sources within the EHR (Epic, Verona, WI) to provide a thorough picture of the patient’s medical record. The EHR includes emergency department, inpatient and outpatient visits, date of visits, reason for visits, admission details, diagnosis codes (ICD-9/ICD-10-CM format), growth measurements, medications prescribed, imaging, laboratory test results, patient problem list, and referrals made. This information is captured and moved into the repository in a structured format. The algorithm was constructed using information from CAG internal and external published or publicly available psychiatric disorder algorithms [11–13, 20–23].

### Inclusion criteria for ADHD cases

ADHD cases were defined by the presence of ICD9 codes beginning with “314.,” *or* ICD10 codes beginning with “F90.,” *or* prescriptions for ADHD-specific medications in the subject’s EHR. To account for the various possible issues in diagnosing ADHD, we required more than one ADHD “hit” at different visits for inclusion. At minimum, the patient must have the following: (1) two separate diagnosis days; (2) two separate ADHD medication prescription days; *(*3) one diagnosis day *and* one ADHD medication day on separate calendar days. Later, we added to the algorithm; (4) one prescription and one result from an abstracted note; or (5) one diagnosis code and one result from an abstracted note. All cases had a diagnosis of ADHD at 4 years or greater, keeping with the American Academy of Pediatrics Clinical Practice Guidelines for ADHD [3].

Keywords were selected to search for ADHD phenotypes (Additional file 1: Table S1). An ADHD medication list was sourced from the eMERGE ADHD algorithm [20], and edited to include medications most likely prescribed to pediatric patients utilizing the online professional version of the Merck Manual, a section on ADHD in Children and Adolescents [24]. We also searched for keywords indicating participation in neurocognitive therapy or psychotherapy.

Free-text notes from a subset of patient charts were reviewed and abstracted by an independent clinical staff member (DA) and analyzed. Abstractions were searched using the ADHD or comorbidity keywords and medications used in the structured search. These results were added to the ADHD and each comorbidity algorithm as further criteria adjustments

### Inclusion criteria for comorbid psychiatric cases

The CAG database was searched for subjects with one or more of nine psychiatric diagnoses: anxiety, autism, major depression, oppositional defiant disorder (ODD), conduct disorder (CD), tic disorders, Tourette syndrome, schizophrenia, and/or bipolar disorder. Subjects with mild/moderate intellectual disability (ID) and learning disabilities (LD) were also included. Algorithms for each condition were created and modified from previously published or publicly available algorithms (see Additional file 1: Tables S2-S12). Generally, each condition was designated at least twice by an IDC9/ICD 10 code on at least two separate visit days. For anxiety, treatment medications designated at least twice or in conjunction with an ICD9/ICD10 code and two separate visit days were used as inclusion criteria; benzodiazepines were not included as they are often prescribed in children for pre- and post-procedural anxiety. The same rule is applied for schizophrenia and bipolar disorder medications. Medication selection was guided by the Mental Health Disorders in Children and Adolescents chapter of the online professional version of the Merck Manual [25] and medications lists sourced from eMERGE algorithms [20]. Like ADHD, we later added to each algorithm: one prescription and one result from an abstracted note or one diagnosis code and one result from an abstracted note. Subjects that were cases of both ADHD and one or more psychiatric disorders were considered comorbid ADHD cases.

### Case exclusions

Because several psychiatric disorders were considered, we used general case exclusion criteria for all subjects. A range of exclusionary diagnoses is listed in Additional file 1: Table S13. These primarily include diagnoses consistent with neuronal damage, neoplasms, infectious diseases affecting the brain, and/or severe and profound intellectual disability, where attention and behavioral problems may be evident but likely to be etiologically distinct. We excluded drugs classified under “cardiovascular agents” or “analgesics” in the EHR to account for drugs, such as clonidine, used to treat non-psychiatric indications. Subjects not meeting the minimal inclusion criteria for ADHD or each of the comorbid psychiatric or related conditions were excluded from the case pool.

### Controls

The control inclusion factor was defined as subjects 8 years old or older. This was to avoid patients with a possible ADHD diagnosis, as the DSM-IV criteria for ADHD age of onset were before 7 years old [26]. Control exclusions specify (1) the medical record excludes any prescriptions for psychiatric, neurological, or related disorders and/or (2) a range of ICD9/ICD10 codes addressing comorbid disorders presenting with psychiatric conditions and any mention of psychiatric disorders (Additional file 1: Table S14). In addition, chromosomal anomalies, genetic syndromes, and other syndromes excluded subjects as controls. Learning disabilities and mild/moderate intellectual disability were not excluded.

### Validation

To establish a gold standard for PPV calculations, we conducted an independent electronic medical record review for random cases that were pulled out by the algorithms to confirm they were “true” cases. The PPVs were calculated for ADHD and psychiatric disorder cases and controls. A random sampling of controls was selected for validation of exclusion criteria. For ADHD validation, we chose subjects extracted by the algorithm but not found in the abstraction list nor verified in the chart abstraction. A random sampling of cases for each psychiatric disorder was chosen for validation. The number of cases with abstraction information available was 4032, and the number of abstractions completed was 741.

### ADHD confidence scoring

Each ADHD subject was given a high, moderate, or low confidence score. The scoring system was based on the source and number of sources indicating an ADHD diagnosis or medication. Subjects received “points” for the number of unique diagnosis or medication days, whether an ADHD phenotype or medication was in the psychological abstraction, and whether the participant had psychotherapy or neurocognitive therapy. The score was not an indicator of disease severity; for example, a high-confidence subject with a high number of diagnoses and prescription days could be a patient with longstanding mild ADHD, stable on their current medication regimen. The score is calculated by the sum of (1) number of unique diagnosis days, (2) number of unique ADHD medication days, (3) whether an ADHD phenotype was located in psych abstraction (0 = absent, 1 = present), (4) whether an ADHD medication was located in psych abstraction (0 = absent, 1 = present), and (5) whether a subject was noted to have psychotherapy or neurocognitive therapy (0 = absent, 1 = present). The number of sources can be from 1 to 5 based on the categories above. High confidence is defined as (1) total score of ≥ 20, (2) total score > 9 and number of sources 4–5, or (3) number of diagnosis days > 9 or number of ADHD medications > 9 and number of sources 2–3. Moderate confidence is defined as (1) subjects not defined in high or low confidence. Low confidence is defined as (1) number of diagnosis days + number of ADHD medications = 2 and total score < 3 or (2) number of diagnosis days = 0 and total score < 10 and number of ADHD medications > 2 and no abstraction sources.

## Results

### ADHD algorithm

A total of 51,293 subjects were extracted from the CAG database based on the study inclusion criteria. Of these, 16,902 subjects were classified as controls, and 10,368 were classified as positive cases for one or more of the 10 psychiatric conditions (Table 1). Sample demographics included age mean and median of 11 years old (standard deviation = 6), 51.4% male, and 52% European American,44% African American, and 4% other race. The remainder (24,023) were excluded based on case and control exclusion criteria. The total number of ADHD positive cases was 5840 (56% of total psych-positive cases, 21% of cases + controls, 11% of the total population of subjects; Additional file 1: Table S16). The percentage of the total population is a bit higher than the number quoted by the CDC (9.4%) for the US population [2]. Adding the abstraction into the ADHD algorithm brought the total number of cases meeting criteria from 5830 to 5840 (Table 2). About half of subjects (49.9%) fell into the moderate confidence category, 20.4% had low confidence criteria, and 29.7% met high confidence level criteria.

**Table 1:** Number of subjects produced by algorithms

Condition	Number of subjects
Total number of extracted subjects	51,293
Psych-negative controls	16,902
Psych-positive cases	10,368
Excluded subjects	24,023

**Table 2:** Comparison of search methods for ADHD cases

Search method	ADHD cases found
ICD codes	4597
ICD codes + phenotype keywords	4597
ICD codes + medication keywords	5830
ICD codes + abstractions	4635
ICD codes + medication keywords + abstractions	5840

### ADHD and comorbidities

**Fig. 2:**
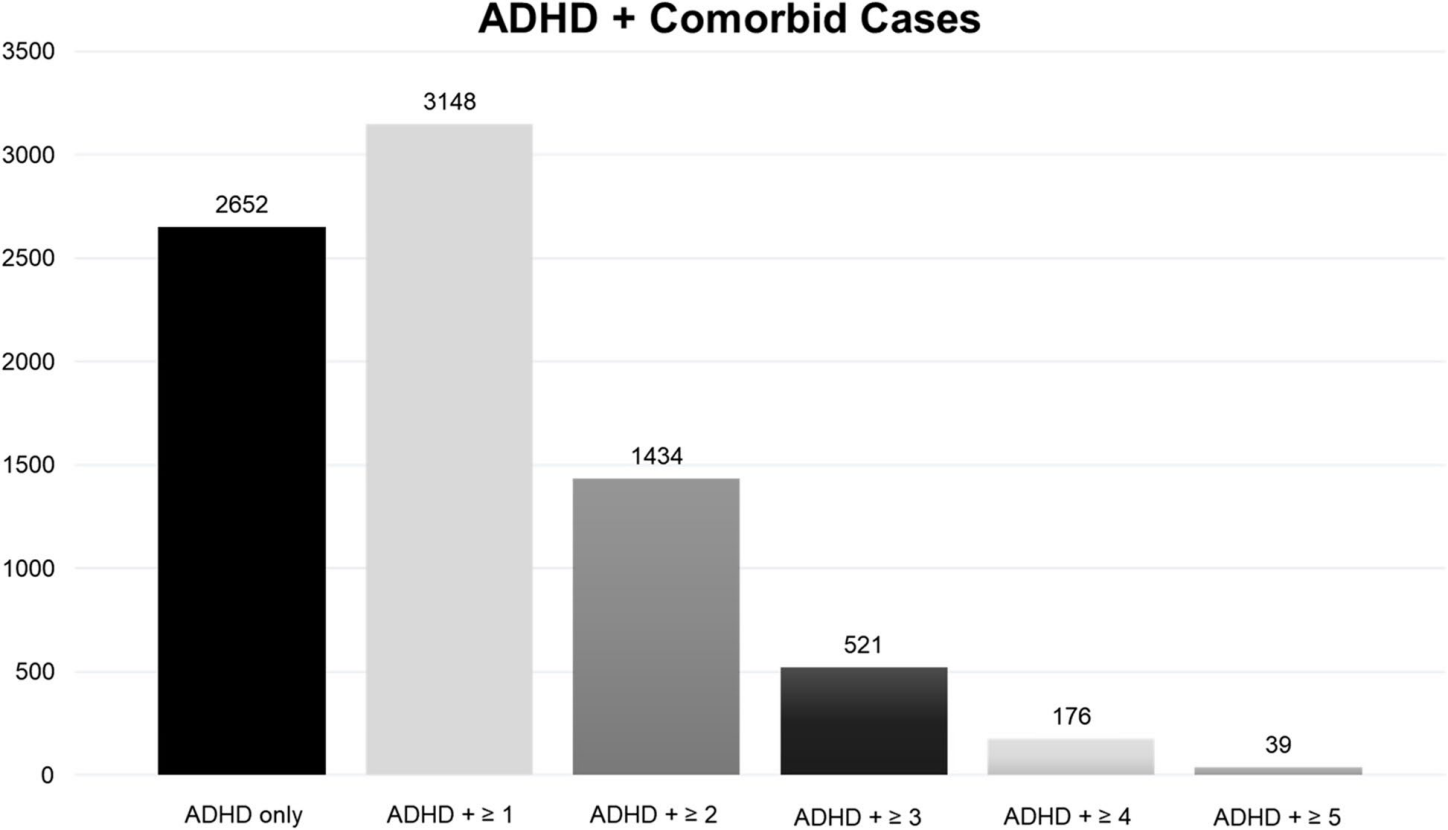
ADHD with all comorbidities. Number of ADHD cases in isolation and with comorbidities

Of the ADHD-positive cases, 46.1% had ADHD alone and 53.9% had one or more comorbidity (Fig. 2). The higher percentage of ADHD with comorbidities has been reported in other studies [2, 6–8]. Anxiety was the most frequent comorbidity observed in our cohort at around 27.1% of ADHD cases (Table 3). Autism presented with ADHD in 15.1% of cases and Tourette syndrome in 1.8% of ADHD cases, which are in line with the results from the 2016 National Survey of Children’s Health (NSCH) [2]. We found lower percentages of ADHD cases with ODD (9.1%), CD (10.1%), and major depression (5.4%), than the NSCH and other studies [8]. Schizophrenia/psychosis was present in 1.2%, bipolar disorder in 2.1%, and tic disorders (including Tourette syndrome) in 3.6% of ADHD cases. All were within the ranges reported in other studies. ADHD cases comorbid with ID were at 3.7% while ADHD presented with LD in 11.8% of cases.

**Table 3:** ADHD with each comorbidity. Each comorbidity number and percentage did not take other comorbidities into consideration

Condition	Number of subjects	Percent (of 5840)
ADHD total	5840	
ADHD in isolation	2692	46.10
ADHD + anxiety	1587	27.17
ADHD + autism	881	15.09
ADHD + conduct disorder	315	5.39
ADHD + oppositional defiant disorder	122	2.09
ADHD + major depression	72	1.23
ADHD + bipolar disorder	592	10.14
ADHD + schizophrenia	529	9.06
ADHD + tics	219	3.75
ADHD + Tourette syndrome	105	1.80
ADHD + intellectual disability	213	3.65
ADHD + learning disability	691	11.83

### Validation of cases and controls

Based on the performance of the algorithms, the PPV for ADHD cases was ~ 95% and for controls was 93% in the subjects extracted from the CAG database. The PPV range for the other psychiatric disorders was 60–100%, with CD and LD having the lowest PPVs based on a random sampling of 15 subjects from each of the 11 ADHD comorbidity combinations (see Additional file 1: Table S17).

## Discussion

Our goal in developing this algorithm was to create and validate an EHR-based algorithm to classify ADHD and comorbid psychiatric status in a pediatric cohort. We used a computerized multi-source phenotyping process for mining EHRs with straightforward implementation that reliably identified ADHD cases, ADHD + comorbid cases, and controls. Given the excellent performance of the algorithm, we recommend it for use in further genomics and other discovery-based studies.

### Algorithm overview

Our algorithm counteracted the fragmented nature of EHR charts by examining multiple sources for evidence to support an ADHD diagnosis. Each subject required multiple positive “hits” to meet the inclusion criteria. While necessitating more evidence of ADHD may yield fewer cases, we viewed this as a worthwhile trade-off for higher confidence of true ADHD and comorbid diagnoses. This process also results in fewer ambiguous cases demanding abstraction for confirmation. Gruschow *et al.* took a similar approach and only performed abstraction validation for subjects with less than three diagnoses [11].

NLP-based phenotyping algorithms have been demonstrated as both sensitive and specific [16]. The primary advantage of NLP is that it allows screening of chart notes for terms and keywords not systematically cataloged in the structured EHR record. We found NLP/text mining did not add any cases, whereas including medications increased the number of cases. Abstractions did not increase the number of cases significantly but did increase the confidence level in many patients.

### Inclusion criteria

We used the inclusion criteria similar to previous studies. The eMERGE algorithm required one diagnosis and one ADHD medication day or two diagnosis days to qualify a case as ADHD [20]. Gruschow *et al.* [11] required only one ADHD diagnosis recorded during an ambulatory visit or hospitalization; Guevara *et al.* [12] had the same criteria but included patients with one or more stimulant prescriptions. Gruschow et al. found ADHD-related keywords or medication prescriptions did not yield additional cases. We also discovered ADHD-related keywords did not increase yield. However, including ADHD-specific medications increased our number of cases by 21%. Within our ADHD cases, 674 subjects had no ICD codes, yet had numerous prescription days. Of these ADHD cases identified only by medication records, 72% were stimulant medications. No other indications of ADHD were found in the abstractions. It is possible that ADHD cases identified only by medication records could have been receiving these ADHD-related medications off-label for a different condition.

### Confidence scoring

Other algorithms have classified the confidence or probability of true ADHD cases in various ways [12, 13]. Daley *et al.* [13] had a weighted confirmation rate system ranging from having a single ADHD diagnosis documented to having a single diagnosis and ADHD criteria met. Guevara and colleagues [12] classified cases as probable, possible, or doubtful. By adding confidence scoring to our results, we allowed for fewer abstractions and false-positive cases.

### Population comparisons

We found 10.76% of our total subjects had ADHD, which is in line with other estimates of prevalence. Population surveys suggest ADHD occurs in most cultures in 5–9% of children [2, 27]. In 2016, the CDC reported the prevalence of ADHD in children and adolescents obtained from the NSCH as 8.4% [2]. Out of a cohort of 15,609, Gruschow *et al.*, [11] found 2030 ADHD cases, or 13%, with their algorithm.

Previous studies examining ADHD with comorbidities have found more patients with additional psychiatric diagnoses than those with ADHD in isolation [2, 7, 8] as we did with our algorithm. Guevara and colleagues [12] identified ADHD cases in 5.2% of their cohort, with 28.7% of those cases having coexisting mental health disorders. Whereas others have reported the highest comorbidities to be ODD and CD [7, 28], anxiety was the most frequent comorbidity observed in our ADHD case population at 26.8%. Our extracted subject population had a lower prevalence of CD and ODD than the US population (1–2% versus 3–10%) which may explain fewer comorbid individuals (see Additional file 1: Table S14). Autism presented with ADHD in around 15% of cases. The anxiety and autism results compare closely to the 2016 NSCH data [2]. The prevalence of comorbidity with major depression was lower than some reports, but higher than others suggesting population biases or different criteria between studies [8, 29]. Learning disabilities have been observed to occur in about 45% of children with ADHD [30], much higher than what we discovered. Our algorithm may have had tighter inclusion criteria for certain psychiatric disorders and learning disabilities than other investigators.

### Limitations

Medical records can become fragmented after transitions in hospital EHRs or when patients seek care at multiple institutions, which can be misleading of a patient’s medical course and true final diagnoses. Although our algorithm is designed to compensate for fragmented patient charts, we did not have access to data outside of our institution. We also faced challenges with medical records housed in systems or paper notes used before the hospital adopted Epic as its EHR.

We were limited by the subjects within the biorepository meeting the study inclusion criteria, and we inadvertently may have selected for subjects not representative of the breadth of the biorepository. Recruitment for the CAG biorepository was not specific for psychiatric disorders. Therefore, our population may look different than others targeting ADHD and psychiatric disorders. We also are uncertain whether ADHD was the primary diagnosis in the cases where a comorbidity was identified. Others have used date ranges between diagnoses to determine this [8, 13] and is something to consider in future iterations of the algorithm.

One limit of simple NLP is the difficulty of distinguishing a negation keyword such as “patient does not have ADHD … .” However, we applied the inclusion criteria requiring multiple instances of a medication or diagnosis to compensate for an abstraction that might be a negation. More advanced NLP using machine learning can account for negations, which are being examined for future algorithms.

Unfortunately, we did not have abstraction data for all subjects, limiting our assessment of the true utility of this component to the algorithm. We did not use it as stand-alone criteria, though abstraction data increased confidence of true cases.

### Future directions

Candidate genes that are likely to be researched within this cohort include the 12 loci associated with ADHD based on GWAS meta-analysis of 20,183 ADHD pediatric cases vs. 35,191 controls (Demontis et al. [31]). In addition, the 4 loci from single-variant analysis and 9 loci from gene-based analysis in 17,149 pediatric and adult cases and 32,411 controls (Rovira et al. [32]). Genes with association to copy number variation in ADHD will be prioritized (Elia et al. [33]). Genes with strong interaction scores with these directly significant genes by pathway or protein-protein interaction will also be queried.

Neuroimaging data such as voxels from functional magnetic resonance imaging (fMRI) will be an exciting new data type to begin quantifying the ADHD and/or other psychiatric disorders as we characterize correlating brain regions.

In terms of treatment improvement of ADHD or other related psychiatric and neurodevelopmental disorders, medication and psychosocial treatment need to be objectively measured for efficacy such as in the Preschool ADHD Treatment Study (PATS) and Multimodal Treatment Study of Children with Attention-Deficit/Hyperactivity Disorder (MTA).

## Conclusion

The advantages of an accurate and high-performing automated algorithm approach to cohort building versus chart review are substantial, particularly with extremely large databases. Chart abstraction required approximately 20–30 min per participant, representing a significant obstacle to scalability. This means the 1079 abstractions would have taken at a minimum about 360 h, or 9 weeks, worth of analyst time. Although the algorithm took several weeks to create, most of this effort was devoted to designing inclusionary/exclusionary criteria, a necessary step for manual processes as well. Establishing an ADHD algorithm that considers comorbidities reduces the amount of time of building and running all comparisons. Critically, subsequent iterations can be run in a matter of minutes, meaning the prospect of keeping pace with an ongoing biorepository is relatively straightforward. These considerations, coupled with high PPVs from validation efforts, urge confidence that the algorithm is a robust and valuable tool for identifying case/control datasets for genetic and discovery-based analyses.

## Supplementary Information

**Additional file 1: Table S1.** ADHD Inclusion/ Exclusion Table. ICD codes and direct terms of ADHD and ADD were used, as well as terms that were specific towards ADHD phenotypes and medications. **Table S2.** Anxiety Inclusion/ Exclusion Table. ICD codes and terms that were specific towards anxiety phenotypes and medications were used. **Table S3.** Autism Inclusion/ Exclusion Table. ICD Codes as well as terms that were specific towards autism phenotypes were used. **Table S4.** Conduct Disorder Inclusion/ Exclusion Table. ICD codes and terms that were specific towards conduct disorder phenotypes were used. **Table S5.** Oppositional Defiant Disorder Inclusion/ Exclusion Table. ICD codes and terms that were specific towards oppositional defiant disorder phenotypes were used. **Table S6.** Major Depressive Disorder Inclusion/ Exclusion Table. ICD codes and terms that were specific towards major depression phenotypes and medications were used. Diagnosis of major depressive disorder was present on at least two (2) distinct calendar days that are at least thirty (30) days apart and not more than one hundred and eighty (180) days apart. **Table S7.** Bipolar Disorder Inclusion/ Exclusion Table. ICD codes and terms that were specific towards bipolar disorder phenotypes and medications were used. Diagnosis of bipolar disorder was present on at least two (2) distinct calendar days that are at least thirty (30) days apart and not more than one hundred and eighty (180) days apart. **Table S8.** Schizophrenia and Psychoses Inclusion/ Exclusion Table. ICD codes and terms that were specific towards schizophrenia and psychoses phenotypes and medications were used. **Table S9.** Tic Disorders Inclusion/ Exclusion Table. ICD codes and terms that were specific towards tic disorder phenotypes were used. **Table S10.** Tourette Syndrome Inclusion/ Exclusion Table. ICD codes and terms that were specific towards Tourette syndrome phenotypes were used. **Table S11.** Intellectual Disability Inclusion/ Exclusion Table. ICD codes and terms that were specific towards intellectual disability phenotypes were used. **Table S12.** Learning Disability Inclusion/ Exclusion Table. ICD codes and terms that were specific towards learning disability phenotypes were used. **Table S13.** Case Exclusion Table. ICD codes and terms used for case exclusions. **Table S14.** Control Exclusion Table. ICD codes and terms used for control exclusions. **Table S15.** Control Syndromes Exclusion Table. ICD codes and terms used for control syndrome exclusions. **Table S16.** Psychiatric Conditions Prevalence in Extracted Subjects. Numbers and percent of psychiatric disorders and comorbidities in all cases and Psych Positive Cases. *has at least of the 10 psychiatric conditions (does not include learning disability or intellectual disability. **Table S17.** Validation of algorithms. Positive Predictive Values (PPV) of algorithms for each psychiatric disorders and comorbidity of ADHD measured.

## Data Availability

The datasets during and/or analyzed during the current study are available from the corresponding author on reasonable request.
