# Supplementary material for: An electronic health record (EHR) phenotype algorithm to identify patients with attention deficit hyperactivity disorders (ADHD) and psychiatric comorbidities
Source: J Neurodev Disord. 2022 Jun 11;14:37. doi: 10.1186/s11689-022-09447-9 (PMC9188139; doi:10.1186/s11689-022-09447-9)
Supplement: Supplementary file 1 — Additional file 1: Table S1. ADHD Inclusion/ Exclusion Table. ICD codes and direct terms of ADHD and ADD were used, as well as terms that were specific towards ADHD phenotypes and medications. Table S2. Anxiety Inclusion/ Exclusion Table. ICD codes and terms that were specific towards anxiety phenotypes and medications were used. Table S3. Autism Inclusion/ Exclusion Table. ICD Codes as well as terms that were specific towards autism phenotypes were used. Table S4. Conduct Disorder Inclusion/ Exclusion Table. ICD codes and terms that were specific towards conduct disorder phenotypes were used. Table S5. Oppositional Defiant Disorder Inclusion/ Exclusion Table. ICD codes and terms that were specific towards oppositional defiant disorder phenotypes were used. Table S6. Major Depressive Disorder Inclusion/ Exclusion Table. ICD codes and terms that were specific towards major depression phenotypes and medications were used. Diagnosis of major depressive disorder was present on at least two (2) distinct calendar days that are at least thirty (30) days apart and not more than one hundred and eighty (180) days apart. Table S7. Bipolar Disorder Inclusion/ Exclusion Table. ICD codes and terms that were specific towards bipolar disorder phenotypes and medications were used. Diagnosis of bipolar disorder was present on at least two (2) distinct calendar days that are at least thirty (30) days apart and not more than one hundred and eighty (180) days apart. Table S8. Schizophrenia and Psychoses Inclusion/ Exclusion Table. ICD codes and terms that were specific towards schizophrenia and psychoses phenotypes and medications were used. Table S9. Tic Disorders Inclusion/ Exclusion Table. ICD codes and terms that were specific towards tic disorder phenotypes were used. Table S10. Tourette Syndrome Inclusion/ Exclusion Table. ICD codes and terms that were specific towards Tourette syndrome phenotypes were used. Table S11. Intellectual Disability Inclusion/ Exclusion Table. ICD codes [file 11689_2022_9447_MOESM1_ESM.docx]

**Title:** An electronic health records (EHR) phenotype algorithm to identify patients with attention deficit hyperactivity disorders (ADHD) and psychiatric comorbidities

**Authors:** Isabella Slaby^a1^, Heather S. Hain^a1^, Debra Abrams^1^, Frank D. Mentch^1^, Patrick M. A. Sleiman^1,2^, Joseph T. Glessner^1^ and Hakon Hakonarson*^1,2^

**Supplementary Tables**

**Supplementary Table 1. ADHD Inclusion/ Exclusion Table.** ICD codes and direct terms of ADHD and ADD were used, as well as terms that were specific towards ADHD phenotypes and medications.

| **Search Category** | **Code** | **Description** |
| --- | --- | --- |
| ICD9 | 314 | Hyperkinetic syndrome of childhood |
| ICD9 | 314.0 | Attention deficit disorder |
| ICD9 | 314.01 | With hyperactivity |
| ICD9 | 314.1 | Hyperkinesis with developmental delay, Developmental disorder of hyperkinesis |
| ICD9 | 314.2 | Hyperkinetic conduct disorder |
| ICD9 | 314.8 | Other specified manifestations of hyperkinetic syndrome |
| ICD9 | 314.9 | Unspecified hyperkinetic syndrome |
| ICD10 | F90 | Hyperkinetic syndrome of childhood |
| ICD10 | F90.0 | Attention-deficit hyperactivity disorder, predominantly inattentive type |
| ICD10 | F90.1 | Attention-deficit hyperactivity disorder, predominantly hyperactive type |
| ICD10 | F90.2 | Attention-deficit hyperactivity disorder, combined type |
| ICD10 | F90.8 | Attention-deficit hyperactivity disorder, other type |
| ICD10 | F90.9 | Attention-deficit hyperactivity disorder, unspecified type |
| Code | %Cardiovascular%Agents% | Exclusion-Medication category |
| Description | Adderall | ADHD Medication |
| Description | Amphetamine | ADHD Medication |
| Description | Aptensio | ADHD Medication |
| Description | Attenade | ADHD Medication |
| Description | Attenta | ADHD Medication |
| Description | Betanamin | ADHD Medication |
| Description | Celgene | ADHD Medication |
| Description | Ceractiv | ADHD Medication |
| Description | Concerta | ADHD Medication |
| Description | Cylert | ADHD Medication |
| Description | Daytrana | ADHD Medication |
| Description | Desoxyn | ADHD Medication |
| Description | Dexedrine | ADHD Medication |
| Description | Dexmethylphenidate | ADHD Medication |
| Description | Dextroamphetamine | ADHD Medication |
| Description | DextroStat | ADHD Medication |
| Description | Evekeo | ADHD Medication |
| Description | Focalin | ADHD Medication |
| Description | Hynidate | ADHD Medication |
| Description | Lisdexamfetamine | ADHD Medication |
| Description | Metadate | ADHD Medication |
| Description | Methylin | ADHD Medication |
| Description | Methyllin | ADHD Medication |
| Description | Methylphenidate | ADHD Medication |
| Description | Pemoline | ADHD Medication |
| Description | Prodrug Amphetamines | ADHD Medication |
| Description | Quillivant | ADHD Medication |
| Description | Rilatine | ADHD Medication |
| Description | Ritalin | ADHD Medication |
| Description | Tradon | ADHD Medication |
| Description | Vyvanse | ADHD Medication |
| Description | Atomoxetine | Neuropsychological Medication |
| Description | Attentin | Neuropsychological Medication |
| Description | Clonidine | Neuropsychological Medication |
| Description | Guanfacine | Neuropsychological Medication |
| Description | Intuniv | Neuropsychological Medication |
| Description | Strattera | Neuropsychological Medication |
| Description | Tomoxetin | Neuropsychological Medication |
| Survey | %adhd%' | ADHD Survey |

**Supplementary Table 2. Anxiety Inclusion/ Exclusion Table.** ICD codes and terms that were specific towards anxiety phenotypes and medications were used.

| **Search Category** | **Code** | **description** |
| --- | --- | --- |
| ICD9 | 300.00_ | Anxiety state, unspecified |
| ICD9 | 300.01_ | Panic disorder without agoraphobia |
| ICD9 | 300.02_ | Generalized anxiety disorder |
| ICD9 | 300.11_ | Conversion disorder |
| ICD9 | 300.20_ | Phobia, unspecified |
| ICD9 | 300.21_ | Agoraphobia with panic disorder |
| ICD9 | 300.22_ | Agoraphobia without mention of panic attacks |
| ICD9 | 300.23_ | Social phobia |
| ICD9 | 300.7_ | Hypochondriasis |
| ICD9 | 313.0_ | Overanxious disorder specific to childhood and adolescence |
| ICD10 | F40.00 | Agoraphobia, unspecified |
| ICD10 | F40.01 | Agoraphobia with panic disorder |
| ICD10 | F40.02 | Agoraphobia without panic disorder |
| ICD10 | F40.10 | Social phobia, unspecified |
| ICD10 | F40.11 | Social phobia, generalized |
| ICD10 | F40.9 | Phobic anxiety disorder, unspecified |
| ICD10 | F41.0 | Panic disorder [episodic paroxysmal anxiety] without agoraphobia |
| ICD10 | F41.1 | Generalized anxiety disorder |
| ICD10 | F41.9 | Anxiety disorder, unspecified |
| ICD10 | F44.4 | Conversion disorder with motor symptom or deficit |
| ICD10 | F44.5 | Conversion disorder with seizures or convulsions |
| ICD10 | F44.6 | Conversion disorder with sensory symptom or deficit |
| ICD10 | F44.7 | Conversion disorder with mixed symptom presentation |
| ICD10 | F45.20 | Hypochondriacal disorder, unspecified |
| ICD10 | F45.21 | Hypochondriasis |
| ICD10 | F45.22 | Body dysmorphic disorder |
| ICD10 | F45.29 | Other hypochondriacal disorders |
| Description | Isocarboxazid | Medication-MAO Inhibitor |
| Description | Moclobemide | Medication-MAO Inhibitor |
| Description | Nardeizine | Medication-MAO Inhibitor |
| Description | Nardil | Medication-MAO Inhibitor |
| Description | Phenelzine | Medication-MAO Inhibitor |
| Description | Tranylcypromine | Medication-MAO Inhibitor |
| Description | Agomelatine | Medication-Other anxiolytic |
| Description | Ambien | Medication-Other anxiolytic |
| Description | Andante | Medication-Other anxiolytic |
| Description | BuSpar | Medication-Other anxiolytic |
| Description | Buspirone | Medication-Other anxiolytic |
| Description | Chlormezanone | Medication-Other anxiolytic |
| Description | Depixol | Medication-Other anxiolytic |
| Description | Equanil | Medication-Other anxiolytic |
| Description | Fluanxol | Medication-Other anxiolytic |
| Description | Flupentixol | Medication-Other anxiolytic |
| Description | Imovane | Medication-Other anxiolytic |
| Description | Luminal | Medication-Other anxiolytic |
| Description | Melitor | Medication-Other anxiolytic |
| Description | Meprobamate | Medication-Other anxiolytic |
| Description | Miltown | Medication-Other anxiolytic |
| Description | Nucloryl | Medication-Other anxiolytic |
| Description | Pedicloryl | Medication-Other anxiolytic |
| Description | Phenobarbital | Medication-Other anxiolytic |
| Description | Sonata | Medication-Other anxiolytic |
| Description | Starnoc | Medication-Other anxiolytic |
| Description | Thymanax | Medication-Other anxiolytic |
| Description | Trancopal | Medication-Other anxiolytic |
| Description | Triclofos | Medication-Other anxiolytic |
| Description | Triclonam | Medication-Other anxiolytic |
| Description | Tricloryl | Medication-Other anxiolytic |
| Description | Tryptophan | Medication-Other anxiolytic |
| Description | Valdoxan | Medication-Other anxiolytic |
| Description | Vanspar | Medication-Other anxiolytic |
| Description | Zaleplon | Medication-Other anxiolytic |
| Description | Zimovane | Medication-Other anxiolytic |
| Description | Zolpidem | Medication-Other anxiolytic |
| Description | Zopiclone | Medication-Other anxiolytic |
| Description | Cymbalta | Medication-Selective Norepinephrine Reuptake Inhibitor |
| Description | Desvenlafaxine | Medication-Selective Norepinephrine Reuptake Inhibitor |
| Description | Duloxetine | Medication-Selective Norepinephrine Reuptake Inhibitor |
| Description | Fetzima | Medication-Selective Norepinephrine Reuptake Inhibitor |
| Description | Levomilnacipran | Medication-Selective Norepinephrine Reuptake Inhibitor |
| Description | Milnacipran | Medication-Selective Norepinephrine Reuptake Inhibitor |
| Description | Pristiq | Medication-Selective Norepinephrine Reuptake Inhibitor |
| Description | Reboxetine | Medication-Selective Norepinephrine Reuptake Inhibitor |
| Description | Savella | Medication-Selective Norepinephrine Reuptake Inhibitor |
| Description | Venlafaxine | Medication-Selective Norepinephrine Reuptake Inhibitor |
| Description | Celexa | Medication-Selective Serotonin Reuptake Inhibitor |
| Description | Cipralex | Medication-Selective Serotonin Reuptake Inhibitor |
| Description | Cipramil | Medication-Selective Serotonin Reuptake Inhibitor |
| Description | Citalopram | Medication-Selective Serotonin Reuptake Inhibitor |
| Description | Dumyrox | Medication-Selective Serotonin Reuptake Inhibitor |
| Description | Escitalopram | Medication-Selective Serotonin Reuptake Inhibitor |
| Description | Faverin | Medication-Selective Serotonin Reuptake Inhibitor |
| Description | Fevarin | Medication-Selective Serotonin Reuptake Inhibitor |
| Description | Floxyfral | Medication-Selective Serotonin Reuptake Inhibitor |
| Description | Fluoxetine | Medication-Selective Serotonin Reuptake Inhibitor |
| Description | Fluvoxamine | Medication-Selective Serotonin Reuptake Inhibitor |
| Description | Lexapro | Medication-Selective Serotonin Reuptake Inhibitor |
| Description | Luvox | Medication-Selective Serotonin Reuptake Inhibitor |
| Description | Paroxetine | Medication-Selective Serotonin Reuptake Inhibitor |
| Description | Paxil | Medication-Selective Serotonin Reuptake Inhibitor |
| Description | Pexeva | Medication-Selective Serotonin Reuptake Inhibitor |
| Description | Prozac | Medication-Selective Serotonin Reuptake Inhibitor |
| Description | Sarafem | Medication-Selective Serotonin Reuptake Inhibitor |
| Description | Sertraline | Medication-Selective Serotonin Reuptake Inhibitor |
| Description | Vibryd | Medication-Selective Serotonin Reuptake Inhibitor |
| Description | Vilazodone | Medication-Selective Serotonin Reuptake Inhibitor |
| Description | Adapin | Medication-Tri/Tetracyclines |
| Description | Allegron | Medication-Tri/Tetracyclines |
| Description | Altapin | Medication-Tri/Tetracyclines |
| Description | Amitriptyline | Medication-Tri/Tetracyclines |
| Description | Amoxapine | Medication-Tri/Tetracyclines |
| Description | Amplit | Medication-Tri/Tetracyclines |
| Description | Anafranil | Medication-Tri/Tetracyclines |
| Description | Anelun | Medication-Tri/Tetracyclines |
| Description | Asendin | Medication-Tri/Tetracyclines |
| Description | Athymil | Medication-Tri/Tetracyclines |
| Description | Aventyl | Medication-Tri/Tetracyclines |
| Description | Bolvidon | Medication-Tri/Tetracyclines |
| Description | Butriptyline | Medication-Tri/Tetracyclines |
| Description | Centrolese | Medication-Tri/Tetracyclines |
| Description | Clomicalm | Medication-Tri/Tetracyclines |
| Description | Clomipramine | Medication-Tri/Tetracyclines |
| Description | Concordin | Medication-Tri/Tetracyclines |
| Description | Depresym | Medication-Tri/Tetracyclines |
| Description | Deprevon | Medication-Tri/Tetracyclines |
| Description | Deprilept | Medication-Tri/Tetracyclines |
| Description | Desipramine | Medication-Tri/Tetracyclines |
| Description | Desyrel | Medication-Tri/Tetracyclines |
| Description | Dopress | Medication-Tri/Tetracyclines |
| Description | Dosulepin | Medication-Tri/Tetracyclines |
| Description | Dothapax | Medication-Tri/Tetracyclines |
| Description | Dothep | Medication-Tri/Tetracyclines |
| Description | Dothiepin | Medication-Tri/Tetracyclines |
| Description | Doxepin | Medication-Tri/Tetracyclines |
| Description | Elavil | Medication-Tri/Tetracyclines |
| Description | Evadene | Medication-Tri/Tetracyclines |
| Description | Evadyne | Medication-Tri/Tetracyclines |
| Description | Evasidol | Medication-Tri/Tetracyclines |
| Description | Galatur | Medication-Tri/Tetracyclines |
| Description | Gamanil | Medication-Tri/Tetracyclines |
| Description | Idom | Medication-Tri/Tetracyclines |
| Description | Imipramine | Medication-Tri/Tetracyclines |
| Description | Iprindole | Medication-Tri/Tetracyclines |
| Description | Irene | Medication-Tri/Tetracyclines |
| Description | Lantanon | Medication-Tri/Tetracyclines |
| Description | Lerivon | Medication-Tri/Tetracyclines |
| Description | Lofepramine | Medication-Tri/Tetracyclines |
| Description | Lomont | Medication-Tri/Tetracyclines |
| Description | Ludiomil | Medication-Tri/Tetracyclines |
| Description | Maprolu | Medication-Tri/Tetracyclines |
| Description | Maprotiline | Medication-Tri/Tetracyclines |
| Description | Maximed | Medication-Tri/Tetracyclines |
| Description | Miansan | Medication-Tri/Tetracyclines |
| Description | Mianserin | Medication-Tri/Tetracyclines |
| Description | Mirtazapine | Medication-Tri/Tetracyclines |
| Description | Molipaxin | Medication-Tri/Tetracyclines |
| Description | Nebril | Medication-Tri/Tetracyclines |
| Description | Noritren | Medication-Tri/Tetracyclines |
| Description | Norpramin | Medication-Tri/Tetracyclines |
| Description | Nortrilen | Medication-Tri/Tetracyclines |
| Description | Nortriptyline | Medication-Tri/Tetracyclines |
| Description | Oleptro | Medication-Tri/Tetracyclines |
| Description | Pamelor | Medication-Tri/Tetracyclines |
| Description | Pertofran | Medication-Tri/Tetracyclines |
| Description | Pertofrane | Medication-Tri/Tetracyclines |
| Description | Pertrofran | Medication-Tri/Tetracyclines |
| Description | Petylyl | Medication-Tri/Tetracyclines |
| Description | Prepadine | Medication-Tri/Tetracyclines |
| Description | Prondol | Medication-Tri/Tetracyclines |
| Description | Prothiaden | Medication-Tri/Tetracyclines |
| Description | Protiaden | Medication-Tri/Tetracyclines |
| Description | Protiadene | Medication-Tri/Tetracyclines |
| Description | Protriptyline | Medication-Tri/Tetracyclines |
| Description | Psymion | Medication-Tri/Tetracyclines |
| Description | Serelan | Medication-Tri/Tetracyclines |
| Description | Silenor | Medication-Tri/Tetracyclines |
| Description | Sinequan | Medication-Tri/Tetracyclines |
| Description | Tertran | Medication-Tri/Tetracyclines |
| Description | Tetramide | Medication-Tri/Tetracyclines |
| Description | Thaden | Medication-Tri/Tetracyclines |
| Description | Tofranil | Medication-Tri/Tetracyclines |
| Description | Tolvin | Medication-Tri/Tetracyclines |
| Description | Tolvon | Medication-Tri/Tetracyclines |
| Description | Trazodone | Medication-Tri/Tetracyclines |
| Description | Trazorel | Medication-Tri/Tetracyclines |
| Description | Trimipramine | Medication-Tri/Tetracyclines |
| Description | Triptil | Medication-Tri/Tetracyclines |
| Description | Trittico | Medication-Tri/Tetracyclines |
| Description | Tymelyt | Medication-Tri/Tetracyclines |
| Description | Vanatrip | Medication-Tri/Tetracyclines |
| Description | Viloxazine | Medication-Tri/Tetracyclines |
| Description | Vivactil | Medication-Tri/Tetracyclines |
| Description | Xerenal | Medication-Tri/Tetracyclines |

**Supplementary Table 3. Autism Inclusion/ Exclusion Table.** ICD Codes as well as terms that were specific towards autism phenotypes were used.

| **Search Category** | **Code** | **Description** |
| --- | --- | --- |
| ICD9 | 299.0 | Autistic disorder |
| ICD9 | 299.8 | Other specified pervasive developmental disorders |
| ICD9 | 299.9 | Unspecified pervasive developmental disorder |
| ICD10 | F84.0 | Autistic disorder |
| ICD10 | F84.5, F84.8 | Other specified pervasive developmental disorders |
| ICD10 | F84.9 | Unspecified pervasive developmental disorder |
| Survey | Y | Autism Survey |

**Supplementary Table 4. Conduct Disorder Inclusion/ Exclusion Table.** ICD codes and terms that were specific towards conduct disorder phenotypes were used.

| **Search Category** | **Code** | **Description** |
| --- | --- | --- |
| ICD9 | 312.0_ | Undersocialized conduct disorder, aggressive type |
| ICD9 | 312.2_ | Socialized conduct disorder |
| ICD9 | 312.39_ | Mixed disturbance of conduct and emotion |
| ICD9 | 312.8_ | Other specified disturbances of conduct not elsewhere classified |
| ICD9 | 312.9_ | Unspecified disturbance of conduct |
| ICD10 | F91.0_ | Conduct disorder confined to family context |
| ICD10 | F91.1_ | Conduct disorder childhood onset type |
| ICD10 | F91.2_ | Conduct disorder adolescent onset type |
| ICD10 | F91.8_ | Other conduct disorders |
| ICD10 | F91.9_ | Conduct disorder, unspecified |

**Supplementary Table 5. Oppositional Defiant Disorder Inclusion/ Exclusion Table.** ICD codes and terms that were specific towards oppositional defiant disorder phenotypes were used.

| **Search Category** | **Code** | **Description** |
| --- | --- | --- |
| ICD9 | 313.81_ | Oppositional defiant disorder |
| ICD10 | F91.3_ | Oppositional defiant disorder |
| Description | %oppositional% | Keyword |
| Description | %ODD% | Keyword |

**Supplementary Table 6. Major Depressive Disorder Inclusion/ Exclusion Table.** ICD codes and terms that were specific towards major depression phenotypes and medications were used. Diagnosis of major depressive disorder was present on at least two (2) distinct calendar days that are at least thirty (30) days apart and not more than one hundred and eighty (180) days apart.

| **Search Category** | **Code** | **Description** |
| --- | --- | --- |
| ICD9 | 296.2 | Major depressive affective disorder, single episode, unspecified |
| ICD9 | 296.21 | Major depressive affective disorder, single episode, mild |
| ICD9 | 296.22 | Major depressive affective disorder, single episode, moderate |
| ICD9 | 296.23 | Major depressive affective disorder, single episode, severe |
| ICD9 | 296.25 | Major depressive affective disorder, single episode, in partial or unspecified remission |
| ICD9 | 296.26 | Major depressive affective disorder, single episode, in full remission |
| ICD9 | 296.3 | Major depressive affective disorder, recurrent episode, unspecified |
| ICD9 | 296.31 | Major depressive affective disorder, recurrent episode, mild |
| ICD9 | 296.32 | Major depressive affective disorder, recurrent episode, moderate |
| ICD9 | 296.33 | Major depressive affective disorder, recurrent episode, severe, without mention of psychotic behavior |
| ICD9 | 296.35 | Major depressive affective disorder, recurrent episode, in partial or unspecified remission |
| ICD9 | 296.36 | Major depressive affective disorder, recurrent episode, in full remission |
| ICD10 | F32.0 | Major depressive disorder, single episode, mild |
| ICD10 | F32.1 | Major depressive disorder, single episode, moderate |
| ICD10 | F32.2 | Major depressive disorder, single episode, severe without psychotic features |
| ICD10 | F32.4 | Major depressive disorder, single episode, in partial remission |
| ICD10 | F32.5 | Major depressive disorder, single episode, in full remission |
| ICD10 | F32.9 | Major depressive disorder, single episode, unspecified |
| ICD10 | F33.0 | Major depressive disorder, recurrent, mild |
| ICD10 | F33.1 | Major depressive disorder, recurrent, moderate |
| ICD10 | F33.2 | Major depressive disorder, recurrent severe without psychotic features |
| ICD10 | F33.40 | Major depressive disorder, recurrent, in remission, unspecified |
| ICD10 | F33.41 | Major depressive disorder, recurrent, in partial remission |
| ICD10 | F33.42 | Major depressive disorder, recurrent, in full remission |
| ICD10 | F33.9 | Major depressive disorder, recurrent, unspecified |
| Description | Amitriptyline | Medication-Tri/Tetracyclines |
| Description | Amoxapine | Medication-Tri/Tetracyclines |
| Description | Bupropion | Medication-Norepinephrine–Dopamine Reuptake Inhibitor |
| Description | Citalopram | Medication-Selective Serotonin Reuptake Inhibitor |
| Description | Clomipramine | Medication-Tri/Tetracyclines |
| Description | Desipramine | Medication-Tri/Tetracyclines |
| Description | Desvenlafaxine | Medication-Selective Norepinephrine Reuptake Inhibitor |
| Description | Doxepin | Medication-Tri/Tetracyclines |
| Description | Duloxetine | Medication-Selective Norepinephrine Reuptake Inhibitor |
| Description | Escitalopram | Medication-Selective Serotonin Reuptake Inhibitor |
| Description | Fluoxetine | Medication-Selective Serotonin Reuptake Inhibitor |
| Description | Fluvoxamine | Medication-Selective Serotonin Reuptake Inhibitor |
| Description | Imipramine | Medication-Tri/Tetracyclines |
| Description | Isocarboxazid | Medication-MAO Inhibitor |
| Description | Maprotiline | Medication-Tri/Tetracyclines |
| Description | Milnacipran | Medication-Selective Norepinephrine Reuptake Inhibitor |
| Description | Mirtazapine | Medication-Tri/Tetracyclines |
| Description | Nefazodone | Medication-Serotonin antagonist and reuptake inhibitor |
| Description | Nomifensine | Medication-Norepinephrine–Dopamine Reuptake Inhibitor |
| Description | Nortriptyline | Medication-Tri/Tetracyclines |
| Description | Paroxetine | Medication-Selective Serotonin Reuptake Inhibitor |
| Description | Phenelzine | Medication-MAO Inhibitor |
| Description | Protriptyline | Medication-Tri/Tetracyclines |
| Description | Selegiline | Medication-MAO Inhibitor |
| Description | Sertraline | Medication-Selective Serotonin Reuptake Inhibitor |
| Description | Tranylcypromine | Medication-MAO Inhibitor |
| Description | Trimipramine | Medication-Tri/Tetracyclines |
| Description | Venlafaxine | Medication-Selective Norepinephrine Reuptake Inhibitor |
| Description | Vilazodone | Medication-Selective Serotonin Reuptake Inhibitor |
| Description | Vortioxetine | Medication-Serotonin Modulator and Stimulator |
| Description | Levomilnacipran | Medication-Selective Norepinephrine Reuptake Inhibitor |
| Description | Elavil | Medication-Tri/Tetracyclines |
| Description | Endep | Medication-Tri/Tetracyclines |
| Description | Amitril | Medication-Tri/Tetracyclines |
| Description | Limbitrol | Medication-Mixed |
| Description | Duo-Vil | Medication-Mixed |
| Description | Etrafon | Medication-Mixed |
| Description | Triavil | Medication-Mixed |
| Description | Asendin | Medication-Tri/Tetracyclines |
| Description | Aplenzin | Medication-Norepinephrine–Dopamine Reuptake Inhibitor |
| Description | Appbutamone | Medication-Norepinephrine–Dopamine Reuptake Inhibitor |
| Description | Budeprion | Medication-Norepinephrine–Dopamine Reuptake Inhibitor |
| Description | Buproban | Medication-Norepinephrine–Dopamine Reuptake Inhibitor |
| Description | Forfivo | Medication-Norepinephrine–Dopamine Reuptake Inhibitor |
| Description | Wellbutrin | Medication-Norepinephrine–Dopamine Reuptake Inhibitor |
| Description | Celexa | Medication-Selective Serotonin Reuptake Inhibitor |
| Description | Anafranil | Medication-Tri/Tetracyclines |
| Description | Norpramin | Medication-Tri/Tetracyclines |
| Description | Pertofrane | Medication-Tri/Tetracyclines |
| Description | Pristiq | Medication-Selective Norepinephrine Reuptake Inhibitor |
| Description | Adapin | Medication-Tri/Tetracyclines |
| Description | Sinequan | Medication-Tri/Tetracyclines |
| Description | Silenor | Medication-Tri/Tetracyclines |
| Description | Cymbalta | Medication-Selective Norepinephrine Reuptake Inhibitor |
| Description | Lexapro | Medication-Selective Serotonin Reuptake Inhibitor |
| Description | Prozac | Medication-Selective Serotonin Reuptake Inhibitor |
| Description | Gaboxetine | Medication-Selective Serotonin Reuptake Inhibitor |
| Description | Rapiflux | Medication-Selective Serotonin Reuptake Inhibitor |
| Description | Sarafem | Medication-Selective Serotonin Reuptake Inhibitor |
| Description | Selfemra | Medication-Selective Serotonin Reuptake Inhibitor |
| Description | Sentroxatine | Medication-Selective Serotonin Reuptake Inhibitor |
| Description | Symbyax | Medication-Mixed |
| Description | Luvox | Medication-Selective Serotonin Reuptake Inhibitor |
| Description | Tofranil | Medication-Tri/Tetracyclines |
| Description | Janimine | Medication-Tri/Tetracyclines |
| Description | Marplan | Medication-MAO Inhibitor |
| Description | Ludiomil | Medication-Tri/Tetracyclines |
| Description | Savella | Medication-Selective Norepinephrine Reuptake Inhibitor |
| Description | Remeron | Medication-Tri/Tetracyclines |
| Description | Serzone | Medication-Serotonin antagonist and reuptake inhibitor |
| Description | Merital | Medication-Norepinephrine–Dopamine Reuptake Inhibitor |
| Description | Alival | Medication-Norepinephrine–Dopamine Reuptake Inhibitor |
| Description | Pamelor | Medication-Tri/Tetracyclines |
| Description | Aventil | Medication-Tri/Tetracyclines |
| Description | Aventyl | Medication-Tri/Tetracyclines |
| Description | Paxil | Medication-Selective Serotonin Reuptake Inhibitor |
| Description | Pexeva | Medication-Selective Serotonin Reuptake Inhibitor |
| Description | Nardil | Medication-MAO Inhibitor |
| Description | Vivactil | Medication-Tri/Tetracyclines |
| Description | Eldepryl | Medication-MAO Inhibitor |
| Description | Zoloft | Medication-Selective Serotonin Reuptake Inhibitor |
| Description | Parnate | Medication-MAO Inhibitor |
| Description | Surmontil | Medication-Tri/Tetracyclines |
| Description | Effexor | Medication-Selective Norepinephrine Reuptake Inhibitor |
| Description | Viibryd | Medication-Selective Serotonin Reuptake Inhibitor |
| Description | Trintellix | Medication-Serotonin Modulator and Stimulator |
| Description | Brintellix | Medication-Serotonin Modulator and Stimulator |
| Description | Fetzima | Medication-Selective Norepinephrine Reuptake Inhibitor |

**Supplementary Table 7. Bipolar Disorder Inclusion/ Exclusion Table.** ICD codes and terms that were specific towards bipolar disorder phenotypes and medications were used. Diagnosis of bipolar disorder was present on at least two (2) distinct calendar days that are at least thirty (30) days apart and not more than one hundred and eighty (180) days apart.

| **Search Category** | **Code** | **Description** |
| --- | --- | --- |
| ICD9 | 296.0_ | Manic disorder, single episode |
| ICD9 | 296.1_ | Manic disorder, recurrent episode |
| ICD9 | 296.4_ | Bipolar affective disorder, manic |
| ICD9 | 296.5_ | Bipolar affective disorder, depressed |
| ICD9 | 296.6_ | Bipolar affective disorder, mixed |
| ICD9 | 296.7 | Bipolar I disorder, most recent episode (or current) unspecified |
| ICD9 | 296.8 | Other and unspecified bipolar disorders |
| ICD9 | 296.80 | Bipolar disorder, unspecified |
| ICD9 | 296.81 | Atypical manic disorder |
| ICD9 | 296.89 | Other bipolar disorders |
| ICD10 | F30_ | Manic episode |
| ICD10 | F30.2 | Manic episode, severe with psychotic symptoms |
| ICD10 | F30.3 | Manic episode in partial remission |
| ICD10 | F30.4 | Manic episode in full remission |
| ICD10 | F30.8 | Other manic episodes |
| ICD10 | F30.9 | Manic episode, unspecified |
| ICD10 | F31_ | Bipolar disorder |
| Description | Lithium | Medication-Mood Stabilizer |
| Description | Eskalith | Medication-Mood Stabilizer |
| Description | Lithobid | Medication-Mood Stabilizer |
| Description | Zyprexa | Medication-Antipsychotic |
| Description | Olanzapine | Medication-Antipsychotic |
| Description | Ziprasidone | Medication-Antipsychotic |
| Description | Geodon | Medication-Antipsychotic |
| Description | Risperidone | Medication-Antipsychotic |
| Description | Risperdal | Medication-Antipsychotic |
| Description | Seroquel | Medication-Antipsychotic |
| Description | Quatipine | Medication-Antipsychotic |
| Description | Abilify | Medication-Antipsychotic |
| Description | Aripiprazole | Medication-Antipsychotic |
| Description | Chlorpromazine | Medication-Antipsychotic |
| Description | Thorazine | Medication-Antipsychotic |
| Description | Lurasidone | Medication-Antipsychotic |
| Description | Latuda | Medication-Antipsychotic |
| Description | Paliperidone | Medication-Antipsychotic |
| Description | Invega | Medication-Antipsychotic |
| Description | Carbamazepine | Medication-Anticonvulsant used for Bipolar |
| Description | Tegretol | Medication-Anticonvulsant used for Bipolar |
| Description | Valproic acid | Medication-Anticonvulsant used for Bipolar |
| Description | Divalproex | Medication-Anticonvulsant used for Bipolar |
| Description | Lamotrigine | Medication-Anticonvulsant used for Bipolar |
| Description | Lamictal | Medication-Anticonvulsant used for Bipolar |

**Supplementary Table 8. Schizophrenia and Psychoses Inclusion/ Exclusion Table.** ICD codes and terms that were specific towards schizophrenia and psychoses phenotypes and medications were used.

| **Search Category** | **Code** | **Description** |
| --- | --- | --- |
| ICD | 295_ | Schizophrenic disorders |
| ICD | 297_ | Delusional disorders |
| ICD | 298_ | Other nonorganic psychoses |
| ICD | 296.04 | Bipolar I disorder, single manic episode, severe, specified as with psychotic behavior |
| ICD | 296.14 | Manic affective disorder, recurrent episode, severe, specified as with psychotic behavior |
| ICD | 296.24 | Major depressive affective disorder, single episode, severe, specified as with psychotic behavior |
| ICD | 296.34 | Major depressive affective disorder, recurrent episode, severe, specified as with psychotic behavior |
| ICD | 296.44 | Bipolar I disorder, most recent episode (or current) manic, severe, specified as with psychotic behavior |
| ICD | 296.54 | Bipolar I disorder, most recent episode (or current) depressed, severe, specified as with psychotic behavior |
| ICD | 296.64 | Bipolar I disorder, most recent episode (or current) mixed, severe, specified as with psychotic behavior |
| ICD | F20_ | Schizophrenia |
| ICD | F21_ | Schizotypal |
| ICD | F22_ | Delusional disorders |
| ICD | F23_ | Brief psychotic disorder |
| ICD | F24_ | Shared psychotic disorder |
| ICD | F25_ | Schizoaffective disorder |
| ICD | F28_ | Other psychotic disorder not due to a substance or known physiological condition |
| ICD | F29_ | Unspecified psychosis not due to a substance or known physiological condition |
| ICD | F30.2 | Manic episode, severe with psychotic symptoms |
| ICD | F31.2 | Bipolar disorder, current episode manic severe with psychotic features |
| ICD | F31.5 | Bipolar disorder, current episode depressed, severe, with psychotic features |
| ICD | F31.64 | Bipolar disorder, current episode mixed, severe, with psychotic features |
| ICD | F32.3 | Major depressive disorder, single episode, severe with psychotic features |
| ICD | F33.3 | Major depressive disorder, recurrent, severe with psychotic symptoms |
| Description | schizo% | Schizophrenia, Schizoaffective, Schizotypal |
| Description | psychosi% | Psychosis |
| Description | psychotic% | Psychotic |
| Description | hallucinat% | Hallucination, Hallucinatory |
| Description | delusion% | Delusions, Delusional |
| Description | Haloperidol | Medication-Antipsychotic |
| Description | Haldol | Medication-Antipsychotic |
| Description | Zyprexa | Medication-Antipsychotic |
| Description | Olanzapine | Medication-Antipsychotic |
| Description | Ziprasidone | Medication-Antipsychotic |
| Description | Geodon | Medication-Antipsychotic |
| Description | Risperidone | Medication-Antipsychotic |
| Description | Risperdal | Medication-Antipsychotic |
| Description | Seroquel | Medication-Antipsychotic |
| Description | Quatipine | Medication-Antipsychotic |
| Description | Abilify | Medication-Antipsychotic |
| Description | Aripiprazole | Medication-Antipsychotic |

**Supplementary Table 9. Tic Disorders Inclusion/ Exclusion Table.** ICD codes and terms that were specific towards tic disorder phenotypes were used.

| **Search Category** | **Code** | **Description** |
| --- | --- | --- |
| ICD9 | 307.2 | Tics |
| ICD10 | F95 | Tic Disorder |
| Description | tic disorder% | Keyword |
| Description | tic % | Keyword |
| Description | % tics % | Keyword |
| Description | %Tourette% | Keyword |

**Supplementary Table 10. Tourette Syndrome Inclusion/ Exclusion Table.** ICD codes and terms that were specific towards Tourette syndrome phenotypes were used.

| **Search Category** | **Code** | **Description** |
| --- | --- | --- |
| ICD9 | 307.23 | Tourette's Disorder |
| ICD10 | F95.2 | Tourette Disorder |
| Description | %Tourette% | Keyword |

**Supplementary Table 11. Intellectual Disability Inclusion/ Exclusion Table.** ICD codes and terms that were specific towards intellectual disability phenotypes were used.

| **Search Category** | **Code** | **Description** |
| --- | --- | --- |
| ICD9 | 317._ | Mild intellectual disabilities |
| ICD9 | 318.0 | Moderate intellectual disabilities |
| ICD9 | 319._ | Unspecified intellectual disabilities |
| ICD9 | 799.52 | Cognitive communication deficit |
| ICD9 | 799.55 | Frontal lobe and executive function deficit |
| ICD9 | 799.59 | Other signs and symptoms involving cognition |
| ICD10 | F70._ | Mild intellectual disabilities |
| ICD10 | F71._ | Moderate intellectual disabilities |
| ICD10 | F78._ | Other intellectual disabilties |
| ICD10 | F79._ | Unspecified intellectual disabilities |
| ICD10 | R41.841 | Cognitive communication deficit |
| ICD10 | R41.844 | Frontal lobe and executive function deficit |
| Description | %intellect%disability% | Keyword |
| Description | %mental%retard% | Keyword |
| Description | %cognitive%deficit% | Keyword |
| Description | %mental%deficit% | Keyword |
| Description | %severe intellect%disability% | Exclusion |
| Description | %profound intellect%disability% | Exclusion |
| Description | %severe mental%retard% | Exclusion |
| Description | %profound mental%retard% | Exclusion |
| Description | %severe cognitive%deficit% | Exclusion |
| Description | %profound cognitive%deficit% | Exclusion |
| Description | %severe mental%deficit% | Exclusion |
| Description | %profound mental%deficit% | Exclusion |

**Supplementary Table 12. Learning Disability Inclusion/ Exclusion Table.** ICD codes and terms that were specific towards learning disability phenotypes were used.

| **Search Category** | **Code** | **Description** |
| --- | --- | --- |
| ICD9 | 315.0 | Developmental reading disorder |
| ICD9 | 315.1 | Mathematics disorder |
| ICD9 | 315.2 | Other specific developmental learning difficulties |
| ICD10 | F81._ | Specific developmental disorders of scholastic skills |
| ICD10 | R48 | Dyslexia and other symbolic dysfunctions, not elsewhere classified |
| Description | %dyslexia%' | Keyword |
| Description | %mathematics% | Keyword |
| Description | %learning% | Keyword |
| Description | %scholastic% | Keyword |
| Description | %academic% | Keyword |
| Description | %mental%retardation% | Exclusion |
| Description | %dev%delay% | Exclusion |
| Description | %add% | Exclusion |
| Description | %motor apraxia% | Exclusion |

**Supplementary Table 13. Case Exclusion Table.** ICD codes and terms used for case exclusions.

| **Search Category** | **Code** | **Description** |
| --- | --- | --- |
| ICD9 | 299.1 | Childhood disintegrative disorder |
| ICD9 | 759.5 | Tuberous sclerosis |
| ICD9 | 006.5 | Amebic brain abscess |
| ICD9 | 013.2 | Tuberculoma of brain |
| ICD9 | 191_ | Malignant neoplasm of brain |
| ICD9 | 192_ | Malignant neoplasm of other and unspecified parts of nervous system |
| ICD9 | 237.7_ | Neurofibromatosis |
| ICD9 | 301.51 | Chronic factitious illness with physical symptoms (Munchausen) |
| ICD9 | 348.1 | Anoxic brain damage |
| ICD9 | 348.2 | Benign intracranial hypertension |
| ICD9 | 348.3 | Encephalopathy, not elsewhere classified |
| ICD9 | 348.4 | Compression of brain |
| ICD9 | 348.5 | Cerebral edema |
| ICD9 | 348.8 | Other conditions of brain |
| ICD9 | 348.9 | Unspecified condition of brain |
| ICD9 | 437.2 | Hypertensive encephalopathy |
| ICD9 | 767_ | Subdural and cerebral hemorrhage |
| ICD9 | 767.9 | Birth trauma, unspecified |
| ICD9 | 800-804 | Fracture of skull |
| ICD9 | 959.01 | Head injury, unspecified |
| ICD9 | 046._ | Slow virus infection of central nervous system |
| ICD9 | 47._ | Meningitis due to enterovirus |
| ICD9 | 191._ | Malignant neoplasm of brain |
| ICD9 | 323.6 | Postinfectious encephalitis |
| ICD9 | 323.9 | Encephalitis, unspec. |
| ICD9 | 348.1 | Anoxic brain damage |
| ICD9 | 348.4 | Compression of brain |
| ICD9 | 348.5 | Cerebral edema |
| ICD9 | 349.8_ | Other specified disorders of nervous system |
| ICD9 | 779.5 | Drug withdrawal syndrome in newborn |
| ICD9 | 094_ | Neurosyphilis |
| ICD9 | 192.0_ | Malignant neoplasm of cranial nerves |
| ICD9 | 192.1_ | Malignant neoplasm of cerebral meninges |
| ICD9 | 323.6_ | Postinfectious encephalitis |
| ICD9 | 323.71_ | Toxic encephalitis and encephalomyelitis |
| ICD9 | 323.72_ | Toxic myelitis |
| ICD9 | 323.9_ | Unspecified causes of encephalitis, myelitis, and encephalomyelitis |
| ICD9 | 854.01-6 | Intracranial injury of other and unspecified nature without mention of open intracranial wound, with no loss of consciousness |
| ICD10 | F84.3 | Childhood disintegrative disorder |
| ICD10 | Q85.1 | Tuberous sclerosis |
| ICD10 | A06.6 | Amebic brain abscess |
| ICD10 | A17.81 | Tuberculoma of brain |
| ICD10 | C71_ | Malignant neoplasm of brain |
| ICD10 | C72_ | Malignant neoplasm of other and unspecified parts of nervous system |
| ICD10 | Q85.0_ | Neurofibromatosis |
| ICD10 | F68.1 | Chronic factitious illness with physical symptoms (Munchausen) |
| ICD10 | G93.1 | Anoxic brain damage |
| ICD10 | G93.2 | Benign intracranial hypertension |
| ICD10 | G93.4 | Encephalopathy, not elsewhere classified |
| ICD10 | G93.5 | Compression of brain |
| ICD10 | G93.6 | Cerebral edema |
| ICD10 | G93.8 | Other conditions of brain |
| ICD10 | G93.9 | Unspecified condition of brain |
| ICD10 | I67.4 | Hypertensive encephalopathy |
| ICD10 | P10.0, P10.1, P10.4, P15.9, P52_ | Subdural and cerebral hemorrhage |
| ICD10 | P15.9 | Birth trauma, unspecified |
| ICD10 | S02.1_-4_, S02.6-9_ | Fracture of skull |
| ICD10 | S09.8_, S09.90 | Head injury, unspecified |
| ICD10 | A81.81, A81.01, A81.00, A81.09, A81.1, A81.2, A81.82, A81.83, A81.89, A81.9 | Slow virus infection of central nervous system |
| ICD10 | A87.0, A87.8, A87.9 | Meningitis due to enterovirus |
| ICD10 | C71.0-C71.9 | Malignant neoplasm of brain |
| ICD10 | G04.00, G04.01, G04.30, G04.31, G04.39, G05.4 | Postinfectious encephalitis |
| ICD10 | G04.90, G04.91 | Encephalitis, unspec. |
| ICD10 | G93.1 | Anoxic brain damage |
| ICD10 | G93.5 | Compression of brain |
| ICD10 | G93.6 | Cerebral edema |
| ICD10 | G96.9 | Other specified disorders of nervous system |
| ICD10 | P96.1, P96.2 | Drug withdrawal syndrome in newborn |
| ICD10 | A50.4_, A52.0_-3_ | Neurosyphilis |
| ICD10 | C72.2_-5_ | Malignant neoplasm of cranial nerves |
| ICD10 | C70_ | Malignant neoplasm of cerebral meninges |
| ICD10 | G04.01 | Postinfectious encephalitis |
| ICD10 | G92 | Toxic encephalitis and encephalomyelitis |
| ICD10 | G92 | Toxic myelitis |
| ICD10 | G04.90-1 | Unspecified causes of encephalitis, myelitis, and encephalomyelitis |
| ICD10 | S06.1_, S06.8_, S06.9_ | Intracranial injury of other and unspecified nature without mention of open intracranial wound, with no loss of consciousness |
| Description | %severe intellect%disability% | Keyword |
| Description | %profound intellect%disability% | Keyword |
| Description | %severe mental%retard% | Keyword |
| Description | %profound mental%retard% | Keyword |
| Description | %severe cognitive%deficit% | Keyword |
| Description | %profound cognitive%deficit% | Keyword |
| Description | %severe mental%deficit% | Keyword |
| Description | %profound mental%deficit% | Keyword |

**Supplementary Table 14. Control Exclusion Table.** ICD codes and terms used for control exclusions.

| **Search Category** | **Code** | **Description** |
| --- | --- | --- |
| survey | data_source = 'scanneddata' | ADHD or Autism Survey |
| survey | (description LIKE '%autism%' OR description LIKE '%adhd%') | ADHD or Autism Survey |
| survey | (description like '%autis%' or description like'%PDD%' or description like '%pervasive%' or description like '%panic attack%' or description like '%hallucinations%' or description like '%therapy%' or (description like '% ODD %' or description like '%adhd%' or description like '%asperg%' or description like '%Aspberger%' or description like '%asberger%' or description like '%%add' or description like '%A.D.D.%' or (description like '% ADD,%' and description not like 'MCADD') or (description like '% ADD %' and description not like '%comment%') or description like '%hears voices%' or description like '%suicid%' or description like '%conduct %' or description like '%obsessive compulsive%' or description like '%oppositional %' or description like '%PTSD%' or description like '%posttraumatic%' or description like '%tourette%' or description like '%depression%' or description like '%anxiety%') | CAG Behavior Survey_complaints |
| survey | "(description like '%autis%' or description like'%PDD%' or description like '%pervasive%' or description like '%panic attack%' or description like '%hallucinations%' or description like '%therapy%' or (description like '% ODD %' or description like '%adhd%' or description like '%asperg%' or description like '%Aspberger%' or description like '%asberger%' or description like '%%add' or description like '%A.D.D.%' or (description like '% ADD,%' and description not like 'MCADD') or (description like '% ADD %' and description not like '%comment%') or description like '%hears voices%' or description like '%suicid%' or description like '%conduct %' or description like '%obsessive compulsive%' description like '%OCD%' or description like '%oppositional %' or description like '%PTSD%' or description like '%posttraumatic%' or description like '%tourette%' or description like '%depression%' or description like '%anxiety%')" | CAG Behavior Survey_behavior |
| Source | data_source not like '%order%' | Exclusion |
| Scource | data_source not like 'surgicalhistory' | Exclusion |
| Code | %Cardiovascular%Agents% | Exclusion |
| Code | %Analgesics% | Exclusion |
| ICD9 | 006.5 | Amebic brain abscess |
| ICD9 | 013.2 | Tuberculoma of brain |
| ICD9 | 191 | Malignant neoplasm of brain |
| ICD9 | 192 | Malignant neoplasm of other and unspecified parts of nervous system |
| ICD9 | 237.7 | Neurofibromatosis |
| ICD9 | ( >= '290' and < '320') | Mental disorders |
| ICD9 | (>= '330' and c< '338') | Hereditary and degenerative diseases of the central nervous system |
| ICD9 | 348 | Other conditions of brain |
| ICD9 | 349 | Other and unspecified disorders of the nervous system |
| ICD9 | 437.2 | Hypertensive encephalopathy |
| ICD9 | 742 | Other congenital anomalies of nervous system |
| ICD9 | 996.2 | Mechanical complication of nervous system device, implant, and graft |
| ICD10 | A06.6 | Amebic brain abscess |
| ICD10 | A17.81 | Tuberculoma of brain |
| ICD10 | C71 | Malignant neoplasm of brain |
| ICD10 | C72 | Malignant neoplasm of other and unspecified parts of nervous system |
| ICD10 | Q85.0 | Neurofibromatosis |
| ICD10 | (>= 'F01' and < 'F49') | Mental disorders |
| ICD10 | F54 | Mental disorders |
| ICD10 | (>= 'F60' and < 'F74') | Mental disorders |
| ICD10 | ( >= 'F78' and < 'F82') | Mental disorders |
| ICD10 | F82 | Mental disorders |
| ICD10 | F84 | Mental disorders |
| ICD10 | ( >= 'F90' and < 'F92') | Mental disorders |
| ICD10 | F93 | Mental disorders |
| ICD10 | F94 | Mental disorders |
| ICD10 | F95 | Mental disorders |
| ICD10 | F98 | Mental disorders |
| ICD10 | F99 | Mental disorders |
| ICD10 | E75 | Hereditary and degenerative diseases of the central nervous system |
| ICD10 | F84 | Hereditary and degenerative diseases of the central nervous system |
| ICD10 | ( >= 'G10' and < 'G14') | Hereditary and degenerative diseases of the central nervous system |
| ICD10 | (>= 'G20' and < 'G27') | Hereditary and degenerative diseases of the central nervous system |
| ICD10 | ( >= 'G30' and < 'G33') | Hereditary and degenerative diseases of the central nervous system |
| ICD10 | (>= 'G90' and < 'G96') | Hereditary and degenerative diseases of the central nervous system |
| ICD10 | G99 | Hereditary and degenerative diseases of the central nervous system |
| ICD10 | G93 | Other conditions of brain |
| ICD10 | G92 | Other and unspecified disorders of the nervous system |
| ICD10 | ( >= 'G96' and < 'G99') | Other and unspecified disorders of the nervous system |
| ICD10 | I67.4 | Hypertensive encephalopathy |
| ICD10 | Q01 | Other congenital anomalies of nervous system |
| ICD10 | Q02 | Other congenital anomalies of nervous system |
| ICD10 | Q03 | Other congenital anomalies of nervous system |
| ICD10 | Q04 | Other congenital anomalies of nervous system |
| ICD10 | Q06 | Other congenital anomalies of nervous system |
| ICD10 | Q07 | Other congenital anomalies of nervous system |
| ICD10 | T85.0 | Mechanical complication of nervous system device, implant, and graft |
| ICD10 | T85.1 | Mechanical complication of nervous system device, implant, and graft |
| ICD10 | T85.610 | Mechanical complication of nervous system device, implant, and graft |
| ICD10 | T85.615 | Mechanical complication of nervous system device, implant, and graft |
| ICD10 | T85.620 | Mechanical complication of nervous system device, implant, and graft |
| ICD10 | T85.625 | Mechanical complication of nervous system device, implant, and graft |
| ICD10 | T85.630 | Mechanical complication of nervous system device, implant, and graft |
| ICD10 | T85.635 | Mechanical complication of nervous system device, implant, and graft |
| ICD10 | T85.690 | Mechanical complication of nervous system device, implant, and graft |
| ICD10 | T85.695 | Mechanical complication of nervous system device, implant, and graft |
| Source | data_source = 'medications' |  |
| Description | Adderall | Neuropsychological Medication |
| Description | Alamon | Neuropsychological Medication |
| Description | Alpha-2 Agonists | Neuropsychological Medication |
| Description | Altruline | Neuropsychological Medication |
| Description | Amfebutamone | Neuropsychological Medication |
| Description | Amphetamine | Neuropsychological Medication |
| Description | Aplenzin | Neuropsychological Medication |
| Description | Aropax | Neuropsychological Medication |
| Description | Atarax | Neuropsychological Medication |
| Description | Aterax | Neuropsychological Medication |
| Description | Atomoxetine | Neuropsychological Medication |
| Description | Attenade | Neuropsychological Medication |
| Description | Attenta | Neuropsychological Medication |
| Description | Attentin | Neuropsychological Medication |
| Description | Beneficat | Neuropsychological Medication |
| Description | Besitran | Neuropsychological Medication |
| Description | Betanamin | Neuropsychological Medication |
| Description | Brisdelle | Neuropsychological Medication |
| Description | Bupropion | Neuropsychological Medication |
| Description | Carbamazepine | Neuropsychological Medication |
| Description | Celgene | Neuropsychological Medication |
| Description | Ceractiv | Neuropsychological Medication |
| Description | Clonazepam | Neuropsychological Medication |
| Description | Clonex | Neuropsychological Medication |
| Description | ClonidineHCl | Neuropsychological Medication |
| Description | Concerta | Neuropsychological Medication |
| Description | Cylert | Neuropsychological Medication |
| Description | Daxid | Neuropsychological Medication |
| Description | Daytrana | Neuropsychological Medication |
| Description | Depakote | Neuropsychological Medication |
| Description | Deprax | Neuropsychological Medication |
| Description | Desirel | Neuropsychological Medication |
| Description | Desoxyn | Neuropsychological Medication |
| Description | Desyrel | Neuropsychological Medication |
| Description | Dexedrine | Neuropsychological Medication |
| Description | Dexmethylphenidate | Neuropsychological Medication |
| Description | DextroStat | Neuropsychological Medication |
| Description | Divalproex | Neuropsychological Medication |
| Description | Duurax | Neuropsychological Medication |
| Description | Eleval | Neuropsychological Medication |
| Description | Elontril | Neuropsychological Medication |
| Description | Epilim | Neuropsychological Medication |
| Description | Equetro | Neuropsychological Medication |
| Description | Equipose | Neuropsychological Medication |
| Description | Eskalith | Neuropsychological Medication |
| Description | Fluoxetine | Neuropsychological Medication |
| Description | Focalin | Neuropsychological Medication |
| Description | Fontex | Neuropsychological Medication |
| Description | Gladem | Neuropsychological Medication |
| Description | Guanfacine | Neuropsychological Medication |
| Description | Hydroxyzine | Neuropsychological Medication |
| Description | Hynidate | Neuropsychological Medication |
| Description | Imipramine | Neuropsychological Medication |
| Description | Implicane | Neuropsychological Medication |
| Description | Kloopin | Neuropsychological Medication |
| Description | Kriadex | Neuropsychological Medication |
| Description | Linotril | Neuropsychological Medication |
| Description | Lisdexamfetamine | Neuropsychological Medication |
| Description | Lithium | Neuropsychological Medication |
| Description | Lithobid | Neuropsychological Medication |
| Description | Lowfin | Neuropsychological Medication |
| Description | Lustral | Neuropsychological Medication |
| Description | Masmoran | Neuropsychological Medication |
| Description | Melipramine | Neuropsychological Medication |
| Description | Mesyrel | Neuropsychological Medication |
| Description | Metadate | Neuropsychological Medication |
| Description | Methylin | Neuropsychological Medication |
| Description | Methyllin | Neuropsychological Medication |
| Description | Methylphenidate | Neuropsychological Medication |
| Description | Molpaxin | Neuropsychological Medication |
| Description | Norepinephrine reuptake inhibitor | Neuropsychological Medication |
| Description | NRI | Neuropsychological Medication |
| Description | Olanzapine | Neuropsychological Medication |
| Description | Oleptro | Neuropsychological Medication |
| Description | Orgatrax | Neuropsychological Medication |
| Description | Paroxetine | Neuropsychological Medication |
| Description | Paxam | Neuropsychological Medication |
| Description | Paxil | Neuropsychological Medication |
| Description | Paxistil | Neuropsychological Medication |
| Description | Pemoline | Neuropsychological Medication |
| Description | Petril | Neuropsychological Medication |
| Description | Pexeva | Neuropsychological Medication |
| Description | Prexaton | Neuropsychological Medication |
| Description | Prodrug Amphetamines | Neuropsychological Medication |
| Description | Prozac | Neuropsychological Medication |
| Description | Quiess | Neuropsychological Medication |
| Description | Ravotril | Neuropsychological Medication |
| Description | Rilatine | Neuropsychological Medication |
| Description | Risperdal | Neuropsychological Medication |
| Description | Risperidone | Neuropsychological Medication |
| Description | Ritalin | Neuropsychological Medication |
| Description | Rivatril | Neuropsychological Medication |
| Description | Rivotril | Neuropsychological Medication |
| Description | Sarafem | Neuropsychological Medication |
| Description | Sealdin | Neuropsychological Medication |
| Description | Sedoran | Neuropsychological Medication |
| Description | Sereupin | Neuropsychological Medication |
| Description | Serivo | Neuropsychological Medication |
| Description | Seroxat | Neuropsychological Medication |
| Description | Sertralin | Neuropsychological Medication |
| Description | Sertralie | Neuropsychological Medication |
| Description | SNRI | Neuropsychological Medication |
| Description | Stimulaton | Neuropsychological Medication |
| Description | Strattera | Neuropsychological Medication |
| Description | Tegretol | Neuropsychological Medication |
| Description | Thombran | Neuropsychological Medication |
| Description | Tofranil | Neuropsychological Medication |
| Description | Tomoxetin | Neuropsychological Medication |
| Description | Tradon | Neuropsychological Medication |
| Description | Tran-Q | Neuropsychological Medication |
| Description | Tranquizine | Neuropsychological Medication |
| Description | TrazodoneHCl | Neuropsychological Medication |
| Description | Trazorel | Neuropsychological Medication |
| Description | Tresleen | Neuropsychological Medication |
| Description | Trialodine | Neuropsychological Medication |
| Description | Trittico | Neuropsychological Medication |
| Description | Vistaril | Neuropsychological Medication |
| Description | Voxra | Neuropsychological Medication |
| Description | Vyvanse | Neuropsychological Medication |
| Description | Wellbutrin | Neuropsychological Medication |
| Description | Zoloft | Neuropsychological Medication |
| Description | Zyban | Neuropsychological Medication |
| Description | Zyprexa | Neuropsychological Medication |
| Description | Aripiprazole | Neuropsychological Medication |
| Description | Abilify | Neuropsychological Medication |
| Description | Zristada | Neuropsychological Medication |
| Description | Ziprasidone | Neuropsychological Medication |
| Description | Geodon | Neuropsychological Medication |
| Description | Chlorpromazine | Neuropsychological Medication |
| Description | Thorazine | Neuropsychological Medication |
| Description | Largactil | Neuropsychological Medication |
| Description | Lurasidone | Neuropsychological Medication |
| Description | Latuda | Neuropsychological Medication |
| Description | Pailperidone | Neuropsychological Medication |
| Description | Invega | Neuropsychological Medication |
| Description | Quetiapine | Neuropsychological Medication |
| Description | Seroquel | Neuropsychological Medication |
| Description | Lamotrigine | Neuropsychological Medication |
| Description | Lamictal | Neuropsychological Medication |
| Description | Citalopram | Neuropsychological Medication |
| Description | Celexa | Neuropsychological Medication |
| Description | Escitalopram | Neuropsychological Medication |
| Description | Lexapro | Neuropsychological Medication |
| Description | Flvoxamine | Neuropsychological Medication |
| Description | Luvox | Neuropsychological Medication |
| Description | Duloxetine | Neuropsychological Medication |
| Description | Cymbalta | Neuropsychological Medication |
| Description | Venlafaxine | Neuropsychological Medication |
| Description | Effexor | Neuropsychological Medication |

**Supplementary Table 15. Control Syndromes Exclusion Table.** ICD codes and terms used for control syndrome exclusions.

| **Search Category** | **Code** | **Description** |
| --- | --- | --- |
| ICD9 | 299.1 | Pervasive developmental disorders |
| ICD9 | 345.4 | Localization-related (focal) (partial) epilepsy and epileptic syndromes with complex partial seizures |
| ICD9 | 345.5 | Localization-related (focal) (partial) epilepsy and epileptic syndromes with simple partial seizures |
| ICD9 | 426.7 | Anomalous atrioventricular excitation |
| ICD9 | 758 | Chromosomal anomalies |
| ICD10 | F84.2 | Rett's syndrome |
| ICD10 | F84.3 | Other childhood disintegrative disorder |
| ICD10 | G40.0 | Localization-related (focal) (partial) idiopathic epilepsy and epileptic syndromes with seizures of localized onset |
| ICD10 | G40.1 | Localization-related (focal) (partial) symptomatic epilepsy and epileptic syndromes with simple partial seizures |
| ICD10 | G40.2 | Localization-related (focal) (partial) symptomatic epilepsy and epileptic syndromes with complex partial seizures |
| ICD10 | I45.6 | Pre-excitation syndrome |
| ICD10 | Q9_. | Chromosomal abnormalities, not elsewhere classified |
| Description | epil%synd | Epilepsy syndromes |
| Description | rett%synd | Genetic syndrome |
| Description | fragi%x%synd | Genetic syndrome |
| Description | corneli%lan%synd | Genetic syndrome |
| Description | apert%synd | Genetic syndrome |
| Description | crouzon%synd | Genetic syndrome |
| Description | gold%har%synd | Syndrome |
| Description | hallerm%streif%synd | Genetic syndrome |
| Description | prad%will%synd | Genetic syndrome |
| Description | pitt%hopk%synd | Genetic syndrome |
| Description | Landau%Kleffner%synd | Genetic syndrome |
| Description | bain%roper%synd | Genetic syndrome |
| Description | dravt%synd | Genetic syndrome |
| Description | angelm%synd | Genetic syndrome |
| Description | pier%robin%synd% | Syndrome |
| Description | san%filipp%synd | Genetic syndrome |
| Description | charge%synd | Genetic syndrome |
| Description | beckw%wiedem%synd | Genetic syndrome |
| Description | melas%synd | Genetic syndrome |

**Supplementary Table 16. Psychiatric Conditions Prevalence in Extracted Subjects.** Numbers and percent of psychiatric disorders and comorbidities in all cases and Psych Positive Cases. *has at least of the 10 psychiatric conditions (does not include learning disability or intellectual disability

| **Condition** | **Number of Subjects** | **Percent of Psych Positive (n= 9753)** | **Percent of All (n= 51,293)** |
| --- | --- | --- | --- |
| All Cases | 51,293 |  |  |
| Psych Positive Cases* | 10,368 |  | 20.21 |
| Psych Negative Cases | 16,902 |  |  |
| ADHD | 5840 | 56.33 | 11.39 |
| Anxiety | 4267 | 41.16 | 8.32 |
| Autism | 2123 | 20.48 | 4.14 |
| Major Depression | 884 | 8.53 | 1.72 |
| Bipolar | 234 | 2.26 | 0.46 |
| Schizophrenia | 169 | 1.63 | 0.33 |
| Conduct Disorder | 1062 | 10.24 | 2.07 |
| Oppositional Defiant Disorder | 653 | 6.30 | 1.27 |
| Tics | 456 | 4.40 | 0.89 |
| Tourette | 169 | 1.63 | 0.33 |
| Intellectual Disability* | 368 | 3.55 | 0.72 |
| Learning Disability* | 947 | 9.13 | 1.85 |

**Supplementary Table 17. Validation of algorithms.** Positive Predictive Values (PPV) of algorithms for each psychiatric disorders and comorbidity of ADHD measured.

| **Condition** | **PPV** |
| --- | --- |
| Autism | 100.0 |
| Schizophrenia | 100.0 |
| Tics | 100.0 |
| Tourette | 93.3 |
| Major Depression | 92.3 |
| Intellectual Disability | 86.7 |
| Oppositional Defiant Disorder | 86.7 |
| Bipolar | 72.7 |
| Learning Disability | 71.4 |
| Anxiety | 58.3 |
| Conduct Disorder | 57.1 |
